# Global HIV/AIDS Medicine

**DOI:** 10.3201/eid1406.080258

**Published:** 2008-06

**Authors:** Jeffrey L. Lennox

**Affiliations:** * Emory University School of Medicine, Atlanta, Georgia, USA

In 1988, Paul Volberding and Merle Sande published the first edition of The Medical Management of AIDS. The 6th and last edition of this authoritative reference was published in 1999, leaving a void on the reference shelves of HIV care providers. Twenty years after the original book, Volberding et al. now offer Global HIV/AIDS Medicine. Their aim is to make this “The first textbook aimed at a comprehensive approach to the management of what is truly a global problem.” The first edition of Global HIV/AIDS Medicine has been extensively revamped from the previous textbook. Three new editors have been added, the text has been expanded from 38 to 71 chapters, and the 135 expert authors have been recruited from throughout the world. 

The text is divided into 6 major sections: Epidemiology and Biology of HIV Infection; Prevention, Diagnosis, and Treatment of HIV Infection; Diseases Associated with HIV Infection; Prevention and Management in Resource-Rich Settings; Prevention and Management in Resource-Poor Settings; and Economic and Social Consequences of the HIV Epidemic. Advances in HIV medicine since the publication of the last edition are extensively reviewed in the first and second sections. The chapter on the molecular biology of HIV provides an excellent overview of how HIV and cellular proteins interact. Current practices in antiretroviral treatment are nicely summarized in chapters 15–18. In the last 2 sections of the book, the authors address problems facing HIV care providers practicing in the developing world. Many new chapters have been written for these sections, including chapters on antiretroviral therapy in resource-poor settings, and malaria and HIV; also included is an updated section on parasitic infections among patients residing in the developing world.

Limitations of this book include the predictable minor duplications and contradictions between chapters written by different authors. Given the tome’s comprehensive nature and global purpose, certain diseases are inevitably given short shrift to limit the likelihood of back strain among readers. Any person hoping to learn about progressive multifocal leukoencephalopathy, biliary tract diseases, outcomes of surgery, or manifestations of thrombotic thrombocytopenic purpura in HIV-infected patients will be disappointed. In addition, the well-written chapter on dermatologic diseases is distinguished by the baffling lack of any images of the manifestations it describes. These shortcomings are dwarfed by the positive aspects of this book.

In summary, this is a magnificent work by a group of expert editors and world-class authors. This volume should be a part of every reference collection and an essential tool for any serious provider of HIV care.

